# Supplementary material for: Early-Life Antibiotic Exposure and Childhood Asthma Trajectories: A National Population-Based Birth Cohort
Source: Antibiotics (Basel). 2023 Feb 3;12(2):314. doi: 10.3390/antibiotics12020314 (PMC9952656; doi:10.3390/antibiotics12020314)
Supplement: Supplementary file 1 [file antibiotics-12-00314-s001.zip › antibiotics-2137485-supplementary.pdf]

Supplementary materials

Table S1. Early-life antibiotic exposure up to 24 months for children whose parents have healthcare cards
Table S2. Association between infant antibiotics and asthma phenotypes by sex

- **Table S2a.** Association between early-life antibiotics and asthma phenotypes by sex
- **Table S2b.** Association between early-life antibiotics and asthma phenotypes by sex for children whose parents had healthcare cards

Table S3. Association between early-life antibiotics and asthma phenotypes by 6, 12, 24 months of use
Table S4. Association between different antibiotics used between 0-24 months and asthma phenotypes
Table S5. Outcome and confounding measures
Table S6. Trajectory shapes and Bayesian information criterion

- **Table S6a.** Trajectory shapes and Bayesian information criterion
- **Table S6b.** Average posterior probability

Figure S1. Directed acyclic graph for the association between early-life antibiotic exposure and childhood asthma.
Figure S2. Asthma trajectories of boys and girls following early-life antibiotic exposure.
Methods S1. DAGitty codes for construction of directed acyclic graph
Methods S2. Trajectory analysis

Table S1. Early-life antibiotic exposure up to 24 months for children whose parents have healthcare cards in LSAC B cohort, Australia

**Sample size, n[a]**	5107
Any antibiotic prescribed for children whose parents have healthcare cards, n (%)	
Yes	778 (68.1)
No	365 (31.9)
Number of antibiotic courses exposed during the period, n (%)	
0	365 (31.9)
1	175 (15.3)
2	141 (12.4)
≥3	462 (40.4)
Class of antibiotics exposed by courses, n (%)	
Beta lactam	
1	220 (32.0)
2	146 (21.2)
3	107 (15.6)
≥4	215 (31.2)
Total	688 (100)
First generation cephalosporin	
1	108 (63.2)
2	35 (20.5)
3	18 (10.5)
≥4	10 (5.8)
Total	171 (100)
Second generation cephalosporin	
1	120 (53.6)
2	55 (24.6)
3	22 (9.8)
≥4	27 (12.0)
Total	224 (100)
Quinolone	
1	1 (100)
Total	1 (100)
Macrolide	
1	102 (67.5)
2	28 (18.5)
3	8 (5.3)
≥4	13 (8.7)
Total	151 (100)
Aminoglycoside	
1	1 (100)
Total	1 (100)
Sulfonamide and Trimethoprim	
1	46 (70.8)
2	7 (10.8)
3	5 (7.6)
≥4	7 (10.8)
Total	65 (100)
Nitroimidazole	
1	16 (88.9)
2	2 (11.1)
Total	18 (100)

Footnote: [a] Percentage is calculated using number of antibiotic exposures as the numerator, and the total number of antibiotic exposure in that class as the denominator, multiplied by 100%.

Table S2a. Association between early-life antibiotics and asthma phenotypes by sex

	Girls (n=2102)			Boys (n=2216)			
Asthma phenotype	n	aRR[a] (95% CI)	p-value	n	aRR[a] (95% CI)	p-value	p-value[b]
Always-low risk	1752	1	reference	1756	1	reference	0.06
Early-resolving Asthma	85	1.02 (0.48–2.18)	0.96	128	1.21 (0.67–2.19)	0.52	
Early-persistent asthma	161	**3.04 (1.84–5.03)**	**< 0.001**	223	**1.59 (1.03–2.44)**	**0.04**	
Late-onset asthma	104	0.93 (0.47–1.87)	0.85	109	**2.03 (1.15–3.60)**	**0.02**	

Abbreviation: n, total number of children in the model, aRR, adjusted risk ratio
[a]Adjusted confounders include maternal smoking, maternal alcohol, maternal education, ethnicity, SEIFA, low birthweight, delivery mode, preterm birth, maternal asthma, rurality, exclusive breastfeeding, viral respiratory infection, number of siblings in household, presence of older sibling in household, maternal antibiotics, childhood obesity.
[b]p-value of chi-square test for interaction between antibiotic exposure and sex

Table S2b. Association between early-life antibiotics and asthma phenotypes by sex for children whose parents had healthcare cards

	Girls (n=411)			Boys (n=440)		
Asthma phenotype	n	aRR[a] (95% CI)	p-value	n	aRR[a] (95% CI)	p-value
Always-low risk	331	1	Reference	348	1	reference
Early-resolving asthma	16	1.48 (0.09–23.36)	0.78	23	1.45 (0.32–6.55)	0.63
Early-persistent asthma	41	1.17 (0.40–3.38)	0.77	45	**3.89 (1.07–14.22)**	**0.04**
Late-onset asthma	23	0.88 (0.19–4.14)	0.87	24	5.05 (0.92–27.82)	0.06

Abbreviation: n, total number of children in the model, aRR, adjusted risk ratio
[a] Adjusted confounders include maternal smoking, maternal alcohol, maternal education, ethnicity, SEIFA, low birthweight, delivery mode, preterm birth, maternal asthma, rurality, exclusive breastfeeding, viral respiratory infection, number of siblings in household, presence of older sibling in household, maternal antibiotics, childhood obesity.
[b]p-value of chi-square test for interaction between antibiotic exposure and sex

Table S3. Association between early-life antibiotics and asthma phenotypes by 6, 12, 24 months of use

Asthma phenotype	n	aRR (95% CI)	p-value	aRR[a] (95% CI)	p-value	p-value[b]
**Up to 6 months**						
Always-low risk	173	1.00 (ref)	1.00 (ref)	1.00 (ref)	1.00 (ref)	
Early-resolving asthma	13	1.25 (0.70–2.24)	0.45	0.63 (0.22–1.79)	0.39	
Early-persistent asthma	29	1.57 (1.05–2.37)	0.03	**1.87 (1.16–3.14)**	**0.02**	
Late-onset asthma	9	0.85 (0.43–1.69)	0.64	0.65 (0.23–1.82)	0.41	
**Between 6 and 12 months**						
Always-low risk	355	1.00 (ref)	1.00 (ref)	1.00 (ref)	1.00 (ref)	
Early-resolving asthma	33	1.63 (1.11–2.40)	0.01	1.12 (0.62–2.03)	0.70	0.99
Early-persistent asthma	72	2.05 (1.55–2.71)	< 0.001	**2.61 (1.81–3.77)**	**< 0.001**	
Late-onset asthma	29	1.40 (0.93–2.10)	0.11	1.45 (0.85–2.50)	0.18	
**Between 12 and 24 months**						
Always-low risk	598	1.00 (ref)	1.00 (ref)	1.00 (ref)	1.00 (ref)	
Early-resolving asthma	51	1.53 (1.10–2.12)	0.01	1.10 (0.68–1.79)	0.71	
Early-persistent asthma	104	1.81 (1.42–2.30)	< 0.001	**1.91 (1.36–2.68)**	**< 0.001**	
Late-onset asthma	47	1.38 (0.99–1.93)	0.06	1.42 (0.90–2.24)	0.13	

Note: Abbreviation: n, total number of children in the model, aRR, adjusted risk ratio
[a]Confounders adjusted include maternal smoking, maternal alcohol, maternal education, ethnicity, SEIFA, low birthweight, delivery mode, preterm birth, maternal asthma, rurality, exclusive breastfeeding, viral respiratory infection, number of siblings in household, presence of older sibling in household, maternal antibiotics, obesity.
[b]p-value of chi-square test for interaction between antibiotic exposure and different class of antibiotic

Table S4. Association between different antibiotics used between 0-24 months and asthma phenotypes.

**Asthma phenotype**	**n**	**RR (95% CI)**	**p-value**	**aRR[a] (95% CI)**	**p-value**	p-value[b]
Beta-lactam other than cephalosporin						
At least one course						
Always-low risk	604	1.00 (ref)	1.00 (ref)	1.00 (ref)	1.00 (ref)	
Early-resolving asthma	105	1.48 (1.07–2.04)	0.02	1.23 (0.78–1.96)	0.38	
Early-persistent asthma	86	1.79 (1.41–2.27)	< 0.001	**2.08 (1.50–2.89)**	**< 0.001**	
Late-onset asthma	45	1.40 (1.01–1.95)	0.04	1.48 (0.95–2.31)	0.09	
First generation cephalosporin						
At least one course						
Always-low risk	122	1.00 (ref)	1.00 (ref)	1.00 (ref)	1.00 (ref)	
Early-resolving asthma	22	1.97 (1.13–3.42)	0.02	1.54 (0.68–3.51)	0.30	
Early-persistent asthma	21	1.66 (1.05–2.61)	0.03	1.61 (0.89–2.90)	0.11	
Late-onset asthma	13	1.28 (0.66–2.47)	0.46	1.37 (0.60–3.11)	0.45	
Second generation cephalosporin						
At least one course						
Always-low risk	161	1.00 (ref)	1.00 (ref)	1.00 (ref)	1.00 (ref)	
Early-resolving asthma	39	1.69 (1.01–2.84)	0.05	1.12 (0.50–2.50)	0.79	0.58
Early-persistent asthma	37	2.46 (1.73–3.50)	< 0.001	**2.66 (1.69–4.20)**	**< 0.001**	
Late-onset asthma	14	2.02 (1.24–3.29)	0.004	1.92 (1.00–3.66)	0.05	
Macrolide						
At least one course						
Always-low risk	130	1.00 (ref)	1.00 (ref)	1.00 (ref)	1.00 (ref)	
Early-resolving asthma	31	1.81 (1.04–3.14)	0.04	1.12 (0.43–2.91)	0.81	
Early-persistent asthma	17	0.81 (0.37–1.76)	0.60	**1.92 (1.06–3.49)**	**0.03**	
Late-onset asthma	8	1.52 (0.97–2.40)	0.07	0.90 (0.32–2.54)	0.84	
Sulfonamide and trimethoprim						
At least one course						
Always-low risk	130	1.00 (ref)	1.00 (ref)	1.00 (ref)	1.00 (ref)	
Early-resolving asthma	31	1.48 (0.59–3.74)	0.41	1.49 (0.43–5.10)	0.53	
Early-persistent asthma	17	1.99 (1.06–3.74)	0.03	**2.19 (0.99–4.85)**	**0.05**	
Late-onset asthma	8	1.48 (0.59–3.74)	0.41	1.05 (0.24–4.51)	0.95	

Note: Abbreviation: n, total number of children in the model, aRR, adjusted risk ratio
[a]Confounders adjusted include maternal smoking, maternal alcohol, maternal education, ethnicity, SEIFA, low birthweight, delivery mode, preterm birth, maternal asthma, rurality, exclusive breastfeeding, viral respiratory infection, number of siblings in household, presence of older sibling in household, maternal antibiotics, obesity.
[b]p-value of chi-square test for interaction between antibiotic exposure and different class of antibiotic

Table S5. Outcome and confounding measures

Measures (source)	LSAC data collection, data derivation	Rationale and variable categorization
**Outcomes**		
Current asthma status (B cohort, wave 4-8)	Current asthma status is formulated based on parent reported doctor's diagnosis (*Has your doctor ever told you that study child has asthma?*); and either a past 12-month asthma medication history (*Has study child taken any medication for asthma in the last 12 months?*) or, symptoms of wheezing in the past 12-months (*In the last 12 months, has study child ever had an illness with wheezing in the chest which lasted for 1 week or more?*).	Current asthma status is categorized as a dichotomous variable (yes/no).
**Confounders**		
Maternal related factors		
Ethnicity (B cohort, wave 1)	Parents 1 and 2 were asked about their country of birth.	Children were considered *Australian* if both parent-1 and 2 were born in Australia, or they were considered as *non-Australian*.
Socioeconomic status (B cohort, wave 1)	This was a census-linked data[a] provided by the Australian Bureau of Statistics Census of Population and Housing. Residential postcodes of parents were collected and categorized into 5 categories of neighborhood disadvantages according to the system developed by the Australian Bureau of Statistics.	Socioeconomic position is a composite variable that consists of parental income, years of education, and occupation status. This variable is available in the LSAC study as SEIFA, 'Socio-Economic Indexes for Areas,' that ranks socioeconomic position as quintiles from one to five, with one being the most disadvantaged and five being the least [1].
Healthcare card (B cohort, wave 1)	Both parents were asked about whether they had received healthcare cards separately, both, or neither.	Receiver of healthcare cards would have to meet criteria based on health and economic needs. Healthcare card usage was categorized as a dichotomous variable (yes/no).
Rurality (B cohort, wave 1)	This was based on the postcodes provided by the parents. Postcodes were classified based on the Australian Geography Standard (2011) into 'Major urban', 'other urban', 'bounded locality', and 'rural balance'. 'Rural balance' was considered as *rural*, and others were all considered as *urban.*	Previous literature has identified urbanization as an environmental risk factor to asthma [2].

Table S5. Outcome and confounding measures (continued)

Measures (source)	LSAC data collection, data derivation	Rationale and variable categorization
Maternal education (B cohort, wave 1)	Parent 1 [c] was asked about the highest year of level of education ranging from secondary school to postgraduate degrees, including certificates and diplomas.	Maternal education levels are implicated to influence offspring childhood asthma risks [3]. Previous studies have categorized maternal education levels as less than year, completed year 12, or greater than year 12[4,5]. Maternal education level is categorized into those with 'less than year 12 education', 'completed year 12 education', 'bachelor's degree or having advanced diploma or certificates,' or a 'postgraduate degree or a graduate diploma or certificate.'
Maternal smoking (B cohort, wave 1)	Parent 1[c] was asked about whether she had smoked during pregnancy. Choices include *yes* or *no*.	Two previous studies have adjusted for maternal alcohol as a confounder[6,7]. Therefore, in this study we adjust for maternal smoking during pregnancy using the self-reported measure by parent 1. Maternal smoking is categorized as a dichotomous variable (yes/no).
Alcohol consumption during pregnancy (B cohort, wave 1)	Parent 1[c] was asked about whether she had alcohol during pregnancy. Choices include *yes* or *no*.	Two previous studies have adjusted for maternal alcohol as a confounder[6,7]. Therefore, in this study we adjust for alcohol consumption during pregnancy using the self-reported measure by parent 1. Maternal alcohol was categorized as a dichotomous variable (yes/no).
Maternal asthma (B cohort, wave 1)	Parent 1 [c] was asked about whether she had taken asthma-related medication during pregnancy. Choices include *yes* or *no*.	Maternal asthma is included as a binary variable to assess the association between maternal asthma and offspring asthma risk.
Delivery method (B cohort, wave 1)	Parent 1 [c] was asked about the study child's delivery method. Choice includes *normal, breech, Cesarean, vacuum extraction, forceps*, and *others.*	Delivery method is a categorical variable split into *vaginal, breech, Cesarean section, vacuum suction, forceps,* and *others.*
Breastfeeding (B cohort, wave 1)	Parent 1 [c] was asked: "How old was child when he/she was first given infant formula or other non-breast milk regularly?" Choices include *hasn't had formula regularly, don't know,* or by the number of *days, weeks*, or *months*.	Breastfeeding is categorized as a binary variable; either "exclusive breast-feeding" or "formula-led feeding" if infant formula was given below 6 months of age [8].
Maternal antibiotic use (B cohort, wave 1)	Parent 1 [c] was asked about whether she had used antibiotics during pregnancy. Choices include *yes* or *no.*	Maternal antibiotic use could be an indicator for poor immune development, which can affect childhood propensities for infections and antibiotic use[6]. Maternal antibiotic use also alters infant gut microbiota compositions and may lead to dysbiosis[9], which has associations with childhood asthma development[10]. Maternal antibiotic use was categorized as a dichotomous variable (yes/no).

Table S5. Outcomes and confounding measures (continued)

Measures (source)	LSAC data collection, data derivation	Rationale and variable categorization
Family related factors		
Other children in household (B cohort, wave 2)	Children were asked about how many children lived in the same household.	As Anthonisen et al. highlights, having siblings in the household is protective against asthma risk in children [11]. The number of siblings in the household which is adjusted during the analysis according to different waves of outcomes.
Presence of older sibling (B cohort, wave 2)	Children were asked about whether they have an elder sibling in the same household.	Presence of older siblings in household added a protective effect on odds of asthma.
Child related factors		
Sex of the child (B cohort, wave 1)	Parent 1 [c] was asked about the sex of the study child. Choices include *male* or *female*.	Asthma prevalence differs between males and females; with a higher prevalence in boys during childhood and this ratio reverses in adulthood [12].
Preterm birth (B cohort, wave 1)	This was a calculated variable[b]; using the infant gestation period and preterm birth was considered if gestation period < 37 weeks.	The binary variable *preterm birth* is categorized according to the WHO report as "birth before completing 37 weeks of gestation"[13].
Low birth weight (B cohort, wave 1)	Parent 1 [c] was asked about the birthweight of the baby. Choice of known birthweight with numerical measures in grams or pounds, or unknown birthweight was provided.	Infant birth weight categories were defined by Stanford Children's health and are categorised accordingly [14]. The categories include babies with birthweight *<2500g*, *2500-2999g*, *3000-3999g*, and *4000-5999g*. Low birthweight is considered as babies with birthweight <2500g[14].
Viral respiratory infection (B cohort, wave 1)	Parent 1 [c] was asked about whether the study child had stayed in a hospital for more than one night due to fever or viral illnesses. Choices include *yes* or *no*.	Viral respiratory infection is a risk factor asthma in childhood due to altered development of lung structure and functions [15,16]. Previous literature has highlighted those viral respiratory infections are associated with more frequent antibiotics exposure [17]. The binary viral respiratory infection variable are adjusted.
Ear infection (B cohort, wave 1)	Parent 1[c] was asked about whether the study child has ongoing problems such as ear infection. Choices include *yes* or *no*.	Ear infection status is categorized as a dichotomous measure. Ear infections were the common infection for children less than 3 years [18].
Other infection (B cohort, wave 1)	Parent 1 was asked about whether the study child has other ongoing infections, excluding hearing, visual problems, developmental delays, eczema, diarrhea/colitis, anemia, ear infection, food and digestive allergies, other illnesses, and other physical disabilities. Choices include *yes* or *no*.	Other infection status is categorized as a dichotomous measure.
Childhood obesity (B cohort, wave 3)	Physical measurements were conducted on the study child at age 4/5 and BMI for age percentile was based on CDC growth charts.	Study shows that asthma in childhood can be directly attributed to childhood obesity [19].

Note: [a]Census-linked data
[b]Infant gestation period was calculated using the child's DOB (date of birth) subtract the number of weeks of gestation.
[c]Not all parent 1 were biological mothers; parent 1 is defined as the parent that knows the child best. In this study, the gender of parent 1 was checked and only females were included to best represent the study child's biological mother.

Table S6a. Trajectory shapes and Bayesian information criterion

Number of group classes	Trajectory shapes[a]	BIC[b]	AIC[c]	Entropy
2	3 3*	-5096.0	-5067.3	0.927
2	2 3	-5094.1	-5068.7	0.926
2	1 3*	-5103.4	-5081.1	0.925
2	2 2	-5090.2	-5067.9	0.926
2	1 1**	-5102.3	-5086.3	0.925
3	3 3 3	-5013.1	-4968.48	0.847
3	2 3 3	-5009.0	-4967.6	0.848
3	1 3 3*	-5004.9	-4966.7	0.848
3	3 2 3	-5036.4	-5026.9	0.831
3	2 2 3	-5009.0	-4967.6	0.848
3	1 2 3	-4999.0	-4963.9	0.852
3	2 2 2	-5006.4	-4971.4	0.839
3	1 1 1*	-5009.2	-4983.8	0.862
3	0 0 0*	-5032.4	-5016.5	0.841
4	3 3 3 3	-4986.9	-4926.4	0.888
4	2 3 3 3	-	-	-
4	1 3 3 3	-5022.3	4968.1	0.781
4	3 2 2 2	-5027.3	-4976.4	0.432
4	2 2 2 2	-5023.2	-4975.4	0.564
4	1 2 1 2*	-5014.8	-4976.4	0.511
4	1 1 1 2*	-4965.0	-4926.80	0.855
**4**	**1 1 1 1****	**-4976.4**	**-4941.3**	**0.872**
4	0 0 0 0	-5040.8	-5018.5	0.561
5	3 3 3 3 3	-5054.93	-4978.5	0.541
5	2 3 3 3 3	-5031.9	-4958.6	0.570
5	1 3 3 3 3	-5034.6	-4964.6	0.533
6	3 3 3 3 3 3	-5075.9	-4983.5	0.349
6	2 3 3 3 3 3	-5024.2	-4935.0	0.508
7	3 3 3 3 3 3 3	-5096.8	-4988.5	0.204

Note: Abbreviations: AIC, Akaike's information criterion; BIC, Bayesian information criterion
[a] Trajectory shapes: 0 = intercept, 1 = linear, 2 = quadratic, 3 = cubic.
We examined the significance of all three parameters (linear, quadratic, and cubic) and dropped the ones that were nonsignificant. That is, we started by including linear, quadratic, and cubic parameters for each trajectory. If the cubic parameter was not significant, we dropped it and tested for a quadratic trajectory. If this was not significant, we dropped it and tested for a linear trajectory. We retained the linear parameter even if this was not significant. After each change we compared the results using the BIC/AIC criteria.
[b] BIC, Bayesian information criterion evaluates a model that best-fits all data points; the model with the highest (least negative) value of BIC is preferred.
[c] AIC, Akaike's information criterion evaluates the relative amount of information lost by a given model; the model with the highest (least negative) value of AIC is preferred.
* one or more groups are significant.
**all parameters are significant ($p < 0.05$).
- Some cells may have missing values due to very small proportion of observations within each group.
In Bold: trajectory shape selected for model and analysis.

Table S6b. Average posterior probability

Trajectory group	Average posterior probability
1 (Early-resolving asthma)	0.7639
2 (Always-low risk)	0.9646
3 (Late-onset asthma)	0.8059
4 (Early-persistent asthma)	0.8568

Figure S1. Directed acyclic graph for the association between early-life antibiotic exposure and childhood asthma.

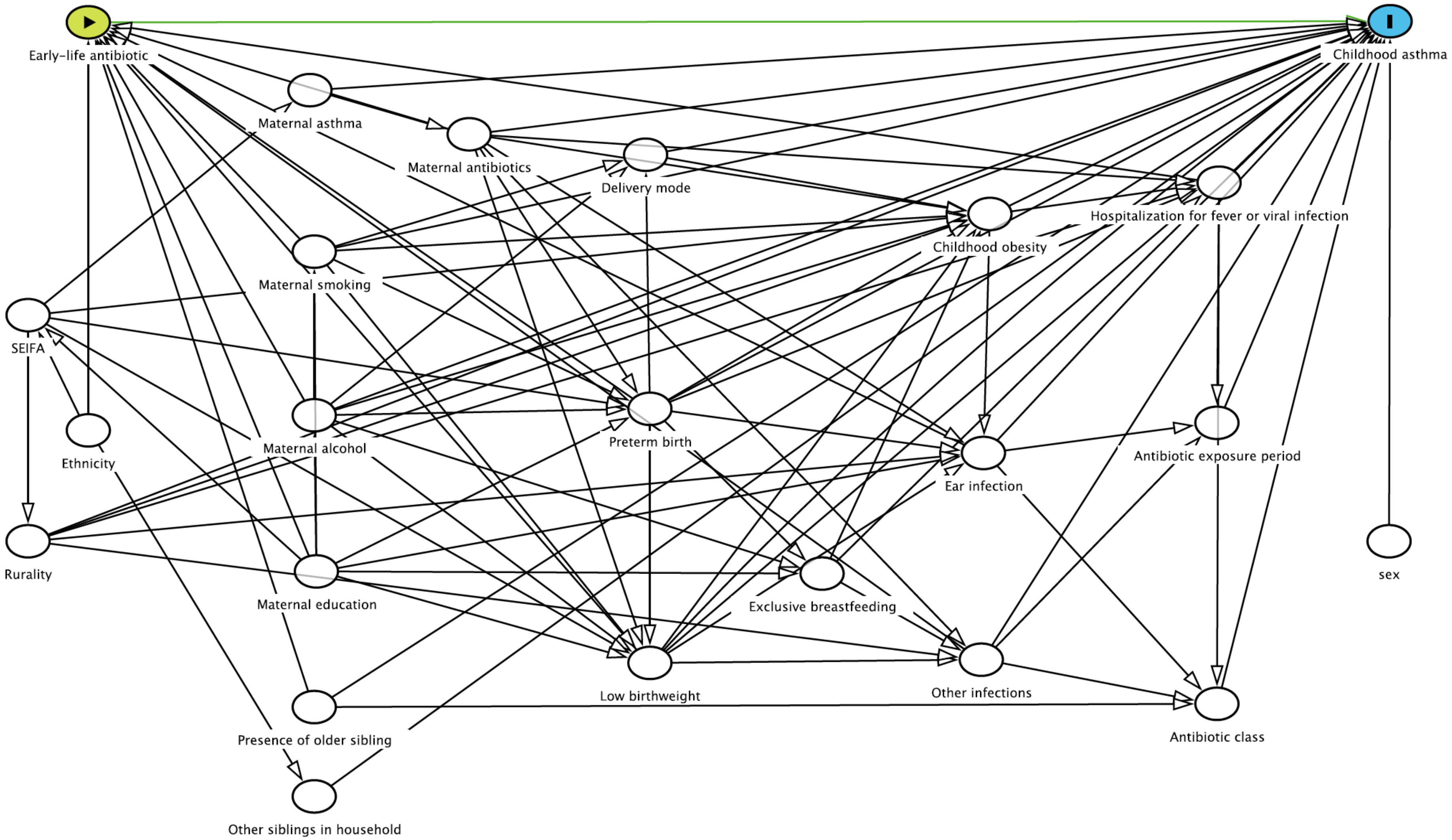

Figure S2. Asthma trajectories of boys and girls following early-life antibiotic exposure.

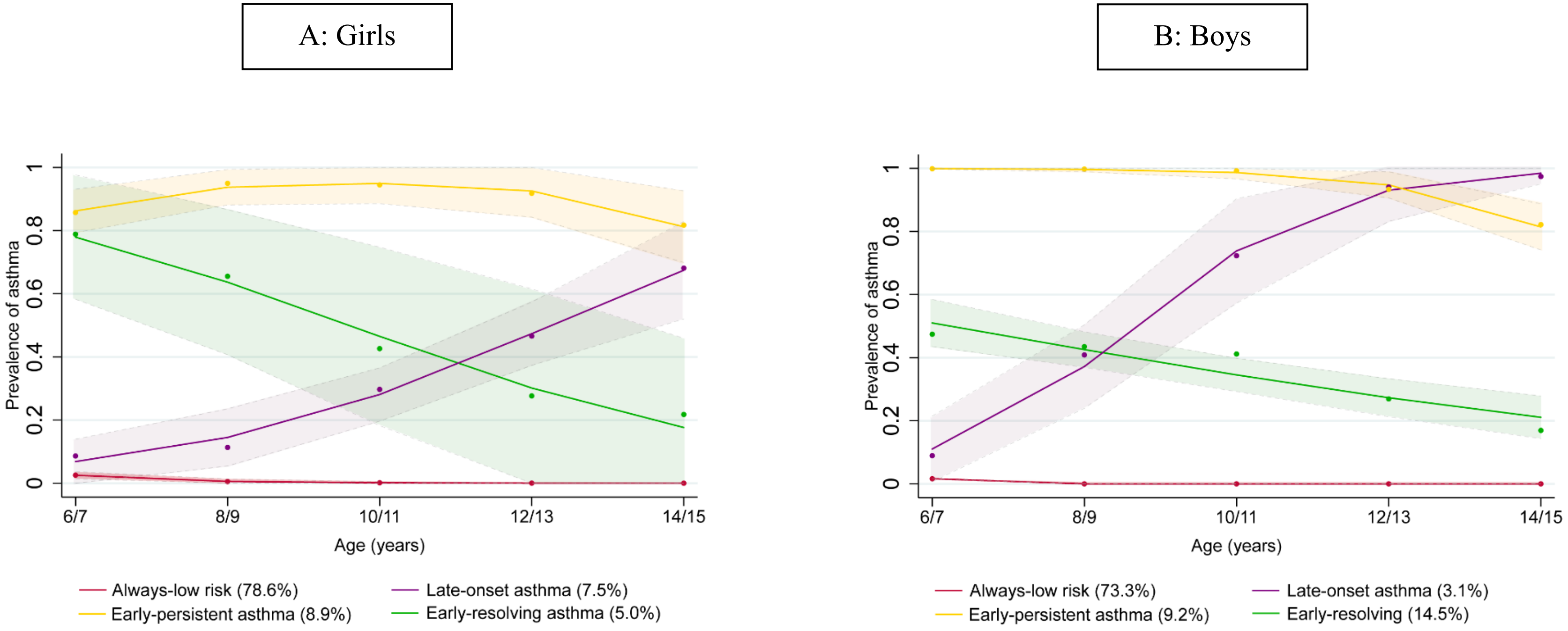

Note: Color bands portray the confidence intervals of the trajectories.

Methods S1. Dagitty codes for construction of directed acyclic graph

```
dag {
"Antibiotic class" [adjusted,pos="0.733,1.181"]
"Antibiotic exposure period" [adjusted,pos="0.733,0.045"]
"Childhood asthma" [outcome,pos="1.044,-1.580"]
"Childhood obesity" [adjusted,pos="0.324,-0.800"]
"Delivery mode" [adjusted,pos="-0.295,-1.040"]
"Ear infection" [adjusted,pos="0.313,0.167"]
"Early-life antibiotic" [exposure,pos="-1.298,-1.576"]
"Exclusive breastfeeding" [adjusted,pos="0.022,0.654"]
"Hospitalization for fever or viral infection" [adjusted,pos="0.737,-0.927"]
"Low birthweight" [adjusted,pos="-0.287,1.016"]
"Maternal alcohol" [adjusted,pos="-0.891,0.014"]
"Maternal antibiotics" [adjusted,pos="-0.613,-1.123"]
"Maternal asthma" [adjusted,pos="-0.899,-1.301"]
"Maternal education" [adjusted,pos="-0.888,0.646"]
"Maternal smoking" [adjusted,pos="-0.891,-0.648"]
"Other infections" [adjusted,pos="0.309,1.003"]
"Other siblings in household" [adjusted,pos="-0.891,1.556"]
"Presence of older sibling" [adjusted,pos="-0.891,1.194"]
"Preterm birth" [adjusted,pos="-0.287,-0.012"]
Ethnicity [adjusted,pos="-1.298,0.075"]
Rurality [adjusted,pos="-1.406,0.524"]
SEIFA [adjusted,pos="-1.406,-0.391"]
sex [adjusted,pos="1.043,0.524"]
"Antibiotic class" -> "Childhood asthma"
"Antibiotic exposure period" -> "Childhood asthma"
"Childhood obesity" -> "Childhood asthma"
"Childhood obesity" -> "Ear infection"
"Childhood obesity" -> "Hospitalization for fever or viral infection"
"Delivery mode" -> "Childhood asthma"
"Delivery mode" -> "Childhood obesity"
"Ear infection" -> "Antibiotic class"
"Ear infection" -> "Antibiotic exposure period"
"Ear infection" -> "Childhood asthma"
"Ear infection" -> "Early-life antibiotic"
"Early-life antibiotic" -> "Childhood asthma"
"Exclusive breastfeeding" -> "Childhood asthma"
"Exclusive breastfeeding" -> "Childhood obesity"
"Exclusive breastfeeding" -> "Other infections"
"Hospitalization for fever or viral infection" -> "Antibiotic class"
"Hospitalization for fever or viral infection" -> "Antibiotic exposure period"
"Hospitalization for fever or viral infection" -> "Childhood asthma"
"Hospitalization for fever or viral infection" -> "Early-life antibiotic"
"Low birthweight" -> "Childhood asthma" [pos="0.289,-0.378"]
"Low birthweight" -> "Childhood obesity"
"Low birthweight" -> "Ear infection"
"Low birthweight" -> "Early-life antibiotic"
"Low birthweight" -> "Hospitalization for fever or viral infection"
"Low birthweight" -> "Other infections"
"Maternal alcohol" -> "Childhood asthma"
"Maternal alcohol" -> "Childhood obesity"
"Maternal alcohol" -> "Delivery mode"
"Maternal alcohol" -> "Early-life antibiotic"
"Maternal alcohol" -> "Exclusive breastfeeding"
"Maternal alcohol" -> "Low birthweight"
"Maternal alcohol" -> "Maternal smoking"
"Maternal alcohol" -> "Preterm birth"
"Maternal antibiotics" -> "Childhood asthma"
"Maternal antibiotics" -> "Childhood obesity"
"Maternal antibiotics" -> "Ear infection"
"Maternal antibiotics" -> "Early-life antibiotic"
"Maternal antibiotics" -> "Hospitalization for fever or viral infection"
"Maternal antibiotics" -> "Low birthweight"
"Maternal antibiotics" -> "Other infections"
"Maternal antibiotics" -> "Preterm birth"
```

```
"Maternal asthma" -> "Childhood asthma"
"Maternal asthma" -> "Maternal antibiotics"
"Maternal education" -> "Ear infection"
"Maternal education" -> "Early-life antibiotic"
"Maternal education" -> "Exclusive breastfeeding"
"Maternal education" -> "Low birthweight"
"Maternal education" -> "Maternal alcohol"
"Maternal education" -> "Maternal smoking"
"Maternal education" -> "Preterm birth"
"Maternal education" -> SEIFA
"Maternal smoking" -> "Childhood asthma"
"Maternal smoking" -> "Childhood obesity"
"Maternal smoking" -> "Delivery mode"
"Maternal smoking" -> "Early-life antibiotic"
"Maternal smoking" -> "Low birthweight"
"Maternal smoking" -> "Preterm birth"
"Other infections" -> "Antibiotic class"
"Other infections" -> "Antibiotic exposure period"
"Other infections" -> "Childhood asthma"
"Other infections" -> "Early-life antibiotic"
"Other siblings in household" -> "Childhood asthma"
"Presence of older sibling" -> "Antibiotic class"
"Presence of older sibling" -> "Childhood asthma"
"Presence of older sibling" -> "Early-life antibiotic"
"Preterm birth" -> "Childhood asthma"
"Preterm birth" -> "Childhood obesity"
"Preterm birth" -> "Delivery mode"
"Preterm birth" -> "Ear infection"
"Preterm birth" -> "Early-life antibiotic"
"Preterm birth" -> "Exclusive breastfeeding"
"Preterm birth" -> "Hospitalization for fever or viral infection"
"Preterm birth" -> "Low birthweight"
Ethnicity -> "Early-life antibiotic"
Ethnicity -> "Other siblings in household"
Ethnicity -> SEIFA
Rurality -> "Childhood asthma"
Rurality -> "Childhood obesity"
Rurality -> "Ear infection"
Rurality -> "Hospitalization for fever or viral infection"
Rurality -> "Other infections"
SEIFA -> "Childhood obesity"
SEIFA -> "Low birthweight"
SEIFA -> "Maternal asthma"
SEIFA -> "Preterm birth"
SEIFA -> Rurality
sex -> "Childhood asthma"
}
```

Methods S2. Trajectory analysis

Rationale for model selection

There are several goodness-of-fit indicators and criteria for the optimal model selection to be based on.

The criteria are listed below:

1. Akaike's information criterion (AIC) and Bayesian information criterion (BIC): value closest to 0 indicates the best-fitting model.
2. Visual graph showing meaningful trajectories.
3. Average posterior probability (AvePP) for each group > 0.7.

First step: selection of the number of trajectories

A trajectory model comprises of the choice of number of groups as well as the order of polynomial of the highest order (quartic). Other goodness-of-fit and criterion were used for order selection of the trajectory model, as we explored 2-, 3-, 4-, 5-, 6-, and 7-group-based trajectory models. The value of the BIC had increased from 2-group to 5-group-based trajectory models and decreased from 5- to 7-group based trajectory models. Within this range, we used the AIC and entropy criterion to select the optimal number groups, the 4-group trajectory model (Table S2a).

Second step: selection of the polynomial terms of each group

We also examined the significance of all three polynomials (linear, quadratic, and cubic) and dropped the ones that were nonsignificant (i.e., p-value > 0.05 for each group). That is, we started by including linear, quadratic, and cubic parameters for each trajectory. If the cubic parameter was not significant, we dropped it and tested for a quadratic trajectory. If this was not significant, we dropped it and tested for a linear trajectory. We retained the linear parameter even if this was not significant. After each change we compared the results using the AIC, BIC, AvePP, and visual inspection of trajectory graph criterion. For example, an AvePP > 0.7 is recommended by Nagin and Odgers [20]. Ultimately, the 4-group trajectory model with order 1,1,1,1 had satisfied all criterion (Table S2b).

References

1. Liao, Z.; Lamb, K.E.; Burgner, D.; Ranganathan, S.; Miller, J.E.; Koplin, J.J.; Dharmage, S.C.; Lowe, A.J.; Ponsonby, A.-L.; Tang, M.L.K.; et al. No obvious impact of caesarean delivery on childhood allergic outcomes: Findings from Australian cohorts. *Arch. Dis. Child.* **2020**, *105*, 664.
2. Morgan, B.W.; Siddharthan, T.; Grigsby, M.R.; Pollard, S.L.; Kalyesubula, R.; Wise, R.A.; Kirenga, B.; Checkley, W. Asthma and Allergic Disorders in Uganda: A Population-Based Study Across Urban and Rural Settings. *J. Allergy Clin. Immunol. Pract.* **2018**, *6*, 1580–1587.e2.
3. Lewis, K.M.; Ruiz, M.; Goldblatt, P.; Morrison, J.; Porta, D.; Forastiere, F.; Hryhorczuk, D.; Zvinchuk, O.; Saurel-Cubizolles, M.-J.; Lioret, S.; et al. Mother's education and offspring asthma risk in 10 European cohort studies. *Eur. J. Epidemiol.* **2017**, *32*, 797–805.
4. Örtqvist, A.K.; Lundholm, C.; Kieler, H.; Ludvigsson, J.F.; Fall, T.; Ye, W.; Almqvist, C. Antibiotics in fetal and early life and subsequent childhood asthma: Nationwide population based study with sibling analysis. *BMJ Br. Med. J.* **2014**, *349*, g6979.
5. Wu, P.; Feldman, A.S.; Rosas-Salazar, C.; James, K.; Escobar, G.; Gebretsadik, T.; Li, S.X.; Carroll, K.N.; Walsh, E.; Mitchel, E.; et al. Relative Importance and Additive Effects of Maternal and Infant Risk Factors on Childhood Asthma. *PLoS ONE* **2016**, *11*, e0151705.
6. Stokholm, J.; Sevelsted, A.; Bønnelykke, K.; Bisgaard, H. Maternal propensity for infections and risk of childhood asthma: A registry-based cohort study. *Lancet Respir. Med.* **2014**, *2*, 631–637.
7. Risnes, K.R.; Belanger, K.; Murk, W.; Bracken, M.B. Antibiotic exposure by 6 months and asthma and allergy at 6 years: Findings in a cohort of 1401 US children. *Am. J. Epidemiol*. **2011**, *173*, 310–318.
8. Greiner, T. Exclusive breastfeeding: measurement and indicators. *Int Breastfeed J.* **2014**, *9*, 18
9. Lamont, R.; Luef, B.M.; Jørgensen, J.S. Childhood inflammatory and metabolic disease following exposure to antibiotics in pregnancy, antenatally, intrapartum and neonatally. *F1000Research* **2020**, *9*, 144.
10. Abrahamsson, T.; Jakobsson, H.E.; Andersson, A.; Björkstén, B.; Engstrand, L.; Jenmalm, M.C. Low gut microbiota diversity in early infancy precedes asthma at school age. *Clin. Exp. Allergy* **2014**, *44*, 842–850.
11. Dik, N.; Tate, R.B.; Manfreda, J.; Anthonisen, N.R. Risk of Physician-Diagnosed Asthma in the First 6 Years of Life. *Chest* **2004**, *126*, 1147–1153.
12. Shah, R.; Newcomb, D.C. Sex Bias in Asthma Prevalence and Pathogenesis. *Front. Immunol.* **2018**, *9*, 2997.
13. WHO. *Born Too Soon, Report*; World Health Organisation: Geneva, Switzerland, 2012.
14. Standord_Medicine. Low Birth Weight: Standard Medicine Children's Health, 2022 Available online: https://www.stanfordchildrens.org/en/topic/default?id=low-birthweight-90-P02382 (accessed on 3 July 2022).
15. Stein, R.T.; Sherrill, D.; Morgan, W.J.; Holberg, C.J.; Halonen, M.; Taussig, L.M.; Wright, A.L.; Martinez, F.D. Respiratory syncytial virus in early life and risk of wheeze and allergy by age 13 years. *Lancet* **1999**, *354*, 541–545.
16. Stern, D.A.; Morgan, W.J.; Halonen, M.; Wright, A.L.; Martinez, F.D. Wheezing and bronchial hyper-responsiveness in early childhood as predictors of newly diagnosed asthma in early adulthood: A longitudinal birth-cohort study. *Lancet* **2008**, *372*, 1058–1064.
17. Nyquist, A.-C.; Gonzales, R.; Steiner, J.F.; Sande, M.A. Antibiotic Prescribing for Children With Colds, Upper Respiratory Tract Infections, and Bronchitis. *JAMA* **1998**, *279*, 875–877.
18. Hu, Y.J.; Wang, J.; Harwell, J.I.; Wake, M. Association of in utero antibiotic exposure on childhood ear infection trajectories: Results from a national birth cohort study. *J. Paediatr. Child Health* **2021**, *57*, 1023–1030.
19. Lang, J.E.; Bunnell, H.T.; Hossain, M.J.; Wysocki, T.; Lima, J.J.; Finkel, T.H.; Bacharier, L.; Dempsey, A.; Sarzynski, L.; Test, M.; et al. Being Overweight or Obese and the Development of Asthma. *Pediatrics* **2018**, *142*, e20182119.
20. Nagin, D.S.; Odgers, C.L. Group-based trajectory modeling in clinical research. *Annu. Rev. Clin. Psychol.* **2010**, *6*, 109–138
